# An improved method for the calculation of unsaturated–saturated water flow by coupling the FEM and FDM

**DOI:** 10.1038/s41598-019-51405-4

**Published:** 2019-10-18

**Authors:** Yulong Gao, Shengyan Pu, Chunmiao Zheng, Shuping Yi

**Affiliations:** 1 School of Environment, Harbin Institute of Technology, Harbin, 150090 China; 2 School of Environmental Science and Engineering, Southern University of Science and Technology, Shenzhen, China; 3 State Key Laboratory of Geohazard Prevention and Geoenvironment Protection (Chengdu University of Technology), Chengdu, Sichuan China; 4 Guangdong Provincial Key Laboratory of Soil and Groundwater Pollution Control, Shenzhen, China

**Keywords:** Hydrogeology, Hydrology

## Abstract

Numerical modeling of water movement in both unsaturated soils and saturated groundwater aquifers is important for water resource management simulations. The development of efficient numerical algorithms for coupling unsaturated and saturated flow has been a long-lasting challenge in hydrologic modeling, especially for regional-scale modeling. In this study, a new method coupling the Finite Element Method (FEM) and Finite Difference Method (FDM), FE-FDM, is developed to solve Richards equation for simulating unsaturated–saturated water flow. The FEM is adopted to discretize the governing equation in the horizontal direction, while the FDM is used in the vertical direction. This method combines the advantages of FEM in domain discretization, especially for complex computational domain, and the advantages of FDM in modeling simplicity and efficiency. The validity of the new method is demonstrated with three test cases that show that the FE-FDM solutions are accurate and are applicable for regional scale problems. In the test cases, the FE-FDM solutions are compared with experimental data and numerical results calculated with common software packages including FEFLOW and COMSOL. This study verified that the FE-FDM is applicable for simulating water flow in the unsaturated–saturated zone.

## Introduction

Understanding the hydrologic cycle is critical for proper management of groundwater resources. In the context of surface-water and groundwater interaction, the hydrologic interactions among soil, vegetation, atmospheric processes, and groundwater dynamics should be considered, which depends to a large extent on the characteristics of the unsaturated zone^1,2^. The commonly used equation for flow in the unsaturated zone, the Richards equation^3,4^, can be troublesome to solve because it is highly nonlinear often causing convergence issues with solution schemes, especially with scale differences between horizontal and vertical dimensions in regional models^1,5^. Because of these difficulties, there are limitations to the coupling of unsaturated and saturated flow in numerical simulations^6^. Therefore, it is essential to develop a mathematical/computational method that can efficiently simulate the coupled unsaturated–saturated flow to manage groundwater resources, especially at the regional scale^2,7^.

The solutions methods for simulating coupled saturated and unsaturated water flow have been studied by many researchers. Rubin^8^ developed a transient numerical model integrating the saturated and unsaturated zones, which solved Richards equation for two-dimensional, transient groundwater flow in a rectangular saturated-unsaturated soil domain. Freeze^9^ further developed a three-dimensional, transient, saturated-unsaturated flow model to solve the saturated-unsaturated flow equation in the unsaturated zone and the saturated flow equation in underlying unconfined and confined aquifers. These early solution methods were prone to mass-balance errors. This issue was investigated by Mitly^10^ and he suggested lumping procedures and methods of time averaging of the storage term to reduce mass-balance errors. Of course, a number of researchers simulate the saturated-unsaturated flow to solve elliptic problems that are robust for high contrasts in material properties and heterogeneity while providing locally conservative velocities for transport over the years^11–17^. However, variably saturated flow simulation has proven challenging^6^.

Since the 1970s, a number of commonly used groundwater flow and transport models have been developed that simulate saturated and unsaturated flow conditions. FEFLOW was first introduced in 1979 by the Institute for Water Resources Planning and Systems Research Inc. (WASY GmbH) of Berlin, Germany a part of The Danish Hydraulic Institute (DHI) group^18^. A 2D finite-element simulation model called SUTRA for saturated-unsaturated, fluid density-dependent groundwater flow with energy transport was developed by Voss^19^. Yeh and Ward^20^ further developed a 3D finite-element model for variably saturated flow, 3DFEMWATER, in 1987. The DHI^21,22^ developed a more comprehensive saturated-unsaturated flow model, based on the FDM, MIKE SHE (Système Hydrologique Européen), where the unsaturated flow is simplified to 1-D in Richards equation while saturated flow is controlled by 3-D Boussinesq equation. COMSOL Multiphysics^23^ is a numerical simulation software based on finite element method which builds models on the basis of general partial differential equations or partial differential equations. Richards equation is built into subsurface flow module of COMSOL to simulate unsaturated-saturated flow. FDM and FEM are recognized as the most popular numerical methods for common groundwater flow models^24^. Although FDM is simpler and more efficient, it is not flexible and efficient for representing irregular boundaries and using refined local grid spacing. FEM requires more computer memory than FDM, though FEM has good mesh adaptability for representing complex boundaries and mesh refinement.

In saturated and unsaturated flow models, the horizontal dimension is generally much larger than the vertical dimension, especially in regional models. In addition, the magnitude of the hydraulic conductivity varies greatly in the vertical direction of the unsaturated zone in the process of rainfall recharge. Thus, a much finer mesh is required in the vertical direction than in the horizontal. Using Different Methods to discrete Equations in horizontal and vertical directions respectively is one of ways to resolve this difference.

This study developed a new numerical method, FE-FDM by coupling FEM and FDM, to solve the Richards equation for simulating unsaturated–saturated water flow. The FEM is adopted to discretize the governing equation in horizontal direction, while the FDM is used in the vertical direction. This method combines the advantages of FEM in domain discretization especially for the irregular boundaries and the advantages of FDM in modeling simplicity and efficiency.

## Numerical form of Richards Equation Discretized by FE-FDM

### Governing equation

The Richards equation, which is commonly used to describe saturated–unsaturated groundwater flow, is expressed as^2,3^:

1 $$\frac{\partial }{\partial x}({K}_{x}(h)\frac{\partial H}{\partial x})+\frac{\partial }{\partial y}({K}_{y}(h)\frac{\partial H}{\partial y})+\frac{\partial }{\partial z}({K}_{z}(h)\frac{\partial H}{\partial z})+w=(C(h)+Se\cdot S)\frac{\partial H}{\partial t}$$

where *Kx*(*h*), *Ky*(*h*), and *Kz*(*h*) are *x*-, *y*-, and *z*-directions hydraulic conductivity as a function of pressure head; *h* is the pressure head; *w* is a source/sink term; *z* is the elevation head; *C*(*h*) is the specific moisture capacity as a function of pressure head; *Se* is the water saturation; *S* is the specific storage; *t* is time; and *H* is hydraulic head calculated by:

2 $$H=h+z$$

The specific moisture capacity *C*(*h*) and the hydraulic conductivity *K*(*h*) both are functions of the pressure head *h* in the governing Eq. (1), which leads equation to be nonlinear. In order to solve the nonlinear governing equation, functional relations must be obtained between two model parameters, *C*(*h*) and *K*(*h*), with unknown variable *h*. Van Genuchten^25^ developed a model for soil water retention curve and hydraulic conductivity function based on earlier work from Mualem^26^.

The saturation-pressure relation is expressed by:

3 $$\theta =\{\begin{array}{cc}{\theta }_{r}-\frac{{\theta }_{s}-{\theta }_{r}}{{(1+{|\alpha h|}^{n})}^{m}} & h < 0\\ {\theta }_{s} & h > 0\end{array}$$

where *θ* is the water content; *θ*s the saturated water content; *θ*r the residual water content; *α*, *m* and *n* are coefficients specifying a particular medium type, and m is restricted as *m* = 1 − 1/*n*.

The hydraulic conductivity function in both saturated and unsaturated soils is written as:

4 $$K(h)=\{\begin{array}{ll}{K}_{s}\cdot S{e}^{1/2}{[1-{(1-S{e}^{\tfrac{1}{m}})}^{m}]}^{2} & h < 0\\ {K}_{s} & h > 0\end{array}$$

where *K*s is the saturated hydraulic conductivity, Se is degree of saturation which could be expressed by:

5 $$Se=\frac{\theta -{\theta }_{r}}{{\theta }_{s}-{\theta }_{r}}$$

The specific moisture capacity can be given by:

6 $$C(h)=\{\begin{array}{ll}{\{\frac{\alpha m}{1-m}({\theta }_{s}-{\theta }_{r})S{e}^{\tfrac{1}{m}}(1-S{e}^{\tfrac{1}{m}})}^{m} & h < 0\\ 0 & h > 0\end{array}$$

### Development of the FE-FDM method

The objective of this section is to obtain the numerical form of Richards equation by FE-FDM. The FEM is used to discretize the governing equation in in horizontal direction while the FDM is used in the vertical direction.

#### Finite element method in the horizontal direction

The governing equation for horizontal flow is discretized by the FEM. According to the Galerkin method, the discrete form in horizontal direction is constructed as follows:

7 $$\begin{array}{c}(C(h)+Se\cdot S)\mathop{\sum }\limits_{i=1}^{Np}({\iint }_{G}{N}_{i}{N}_{j}dxdy)\frac{d{H}_{i}^{k}}{dt}+\mathop{\sum }\limits_{i=1}^{Np}({\iint }_{G}(Kx(h)\frac{\partial {N}_{i}}{\partial x}\frac{\partial {N}_{j}}{\partial x}+Kx(h)\frac{\partial {N}_{i}}{\partial x}\frac{\partial {N}_{j}}{\partial x})dxdy){H}_{i}^{k}\\ -\mathop{\sum }\limits_{i=1}^{Np}({\iint }_{G}{N}_{i}{N}_{j}dxdy)\frac{\partial }{\partial z}({K}_{z}(h)\frac{\partial {H}_{i}^{k}}{\partial z})={\int }_{L}q{N}_{j}ds+{\iint }_{G}w{N}_{j}dxdy\end{array}$$

where *N*_p_ is node number in a horizontal layer; *N*_i_, *N*_j_ is basis function which is a function of *x* and *y*; *k* stands the kth horizontal layer; *q* is specified flux in the boundary.

Symbols were introduced as follows:

8 $${M}_{i,j}=(C(h)+Se\cdot S)\mathop{\sum }\limits_{i=1}^{Np}({\iint }_{G}{N}_{i}{N}_{j}dxdy)$$

9 $${G}_{i,j}=\mathop{\sum }\limits_{i=1}^{Np}({\iint }_{G}(Kx(h)\frac{\partial {N}_{i}}{\partial x}\frac{\partial {N}_{j}}{\partial x}+Kx(h)\frac{\partial {N}_{i}}{\partial x}\frac{\partial {N}_{j}}{\partial x})dxdy)$$

10 $${A}_{i,j}=-\mathop{\sum }\limits_{i=1}^{Np}({\iint }_{G}{N}_{i}{N}_{j}dxdy)$$

11 $${F}_{j}={\int }_{L}q{N}_{j}ds+{\iint }_{G}w{N}_{j}dxdy$$

Then the Eq. (7) is expressed as:

12 $${M}_{i,j}\frac{d{H}_{i}^{k}}{dt}-{A}_{i,j}\frac{\partial }{\partial z}({K}_{z}(h)\frac{\partial {H}_{i}^{k}}{\partial z})+{G}_{i,j}{H}_{i}^{k}={F}_{j}^{k}$$

#### Finite difference method in the vertical direction

In this section, Eq. (12) is discretized by FDM in terms of the space (z direction) and time. A central difference method and a fully implicit difference method are developed for the z- direction and time, respectively.

Equation (12) discretized in the z direction is expressed as:

13 $${M}_{i,j}\frac{d{H}_{i}^{k}}{dt}-{A}_{i,j}\frac{({K}_{z}^{k-\tfrac{1}{2}}(h){H}_{i}^{k-1}-({K}_{z}^{k-\tfrac{1}{2}}(h)+{K}_{z}^{k+\tfrac{1}{2}}(h)){H}_{i}^{k}+{K}_{z}^{k+\tfrac{1}{2}}(h){H}_{i}^{k+1})}{\varDelta {z}^{2}}+{G}_{i,j}{H}_{i}^{k}={F}_{j}^{k}$$

where n is nth time step; ${K}_{z}^{k-\tfrac{1}{2}}$ is harmonic mean of hydraulic conductivity in (*k*-1)th layer and kth layer; ${K}_{z}^{k+\tfrac{1}{2}}$ is harmonic mean of hydraulic conductivity in kth layer and (*k* + 1)th layer.

The method of full implicit discretization is adopted in time discretization. Then the expression is obtained as:

14 $$\begin{array}{c}{M}_{i,j}\frac{{H}_{i}^{k,\,n+1}-{H}_{i}^{k,\,n}}{\Delta t}-{A}_{i,j}\frac{({K}_{z}^{k-\tfrac{1}{2}}(h){H}_{i}^{k-1,\,n+1}-({K}_{z}^{k-\tfrac{1}{2}}(h)+{K}_{z}^{k+\tfrac{1}{2}}(h)){H}_{i}^{k,\,n+1}+{K}_{z}^{k+\tfrac{1}{2}}(h){H}_{i}^{k+1,\,n+1})}{\Delta {z}^{2}}\\ \,+\,{G}_{i,j}{H}_{i}^{k,\,n+1}={F}_{j}^{k}\end{array}$$

According to Eq. (14), the final global equation of the system is formed assembling all the horizontal layers.

15 $$DH=F$$

where *D* is global matrix consisting of the coefficients in the Eq. (14); *H* is the unknown hydraulic head values in the nodes for the current time step and current iteration level; *F* is the known values in the nodes from the last time step and last iteration level.

Equation (15) is a non-linear equation which is solved by Picard iteration.

## Verification Examples

In this study, three test problems are used to verify the accuracy and reliability of the method. The results calculated by FE-FDM are compared with popular models and empirical data.

### Test problem 1: 2D unsaturated-saturated water flow

**Figure 1:**
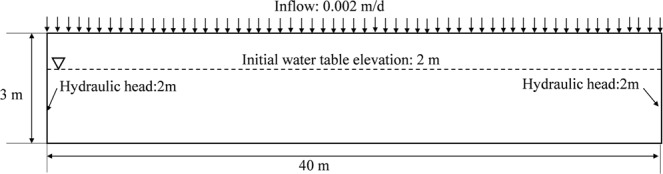
The model of test problem 1.

In this test problem, the study domain is a 2D rectangular unconfined aquifer between two rivers 40 m apart with a 3 m thick aquifer. The soil parameters are presented in the Table 1. The conceptual model of test problem 1 is shown in Fig. 1. The initial hydraulic head is 2 m. The recharge flux is 0.002 m/d at the top boundary. The hydraulic head is 2 m at the left and right boundaries, which are constant head boundaries. The bottom boundary is impermeable.

**Table 1:** Soil parameters of test problem 1.

Ks (m/d)	n (1)	α (1 /m)	θs (1)	θr (1)
0.5	2	0.1	0.3	0

FE-FDM and FEFLOW are used to solve this test problem to calculate both a steady-state solution and a transient solution. FEFLOW, based on the FEM, is widely used in unsaturated-saturated flow simulation. The same rectangular mesh is used for both FE-FDM and FEFLOW with the mesh spacing of 0.5 m in the horizontal direction and 0.3 m in the vertical direction. The domain is discretized with 40 rows and 10 columns of nodes.

**Figure 2:**
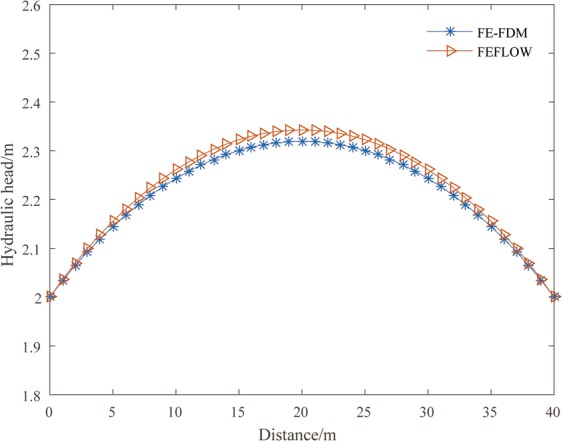
Comparison the water heads distribution calculated by FE-FDM and FEFLOW.

The steady-state results are described first. The steady-state water tables calculated with FE-FDM and FEFLOW are shown on Fig. 2. The water table elevations calculated with FE-FDM are a little lower than that calculated with FEFLOW. The biggest difference in the water table elevations between FE-FDM and FEFLOW is 0.023 m.

**Figure 3:**
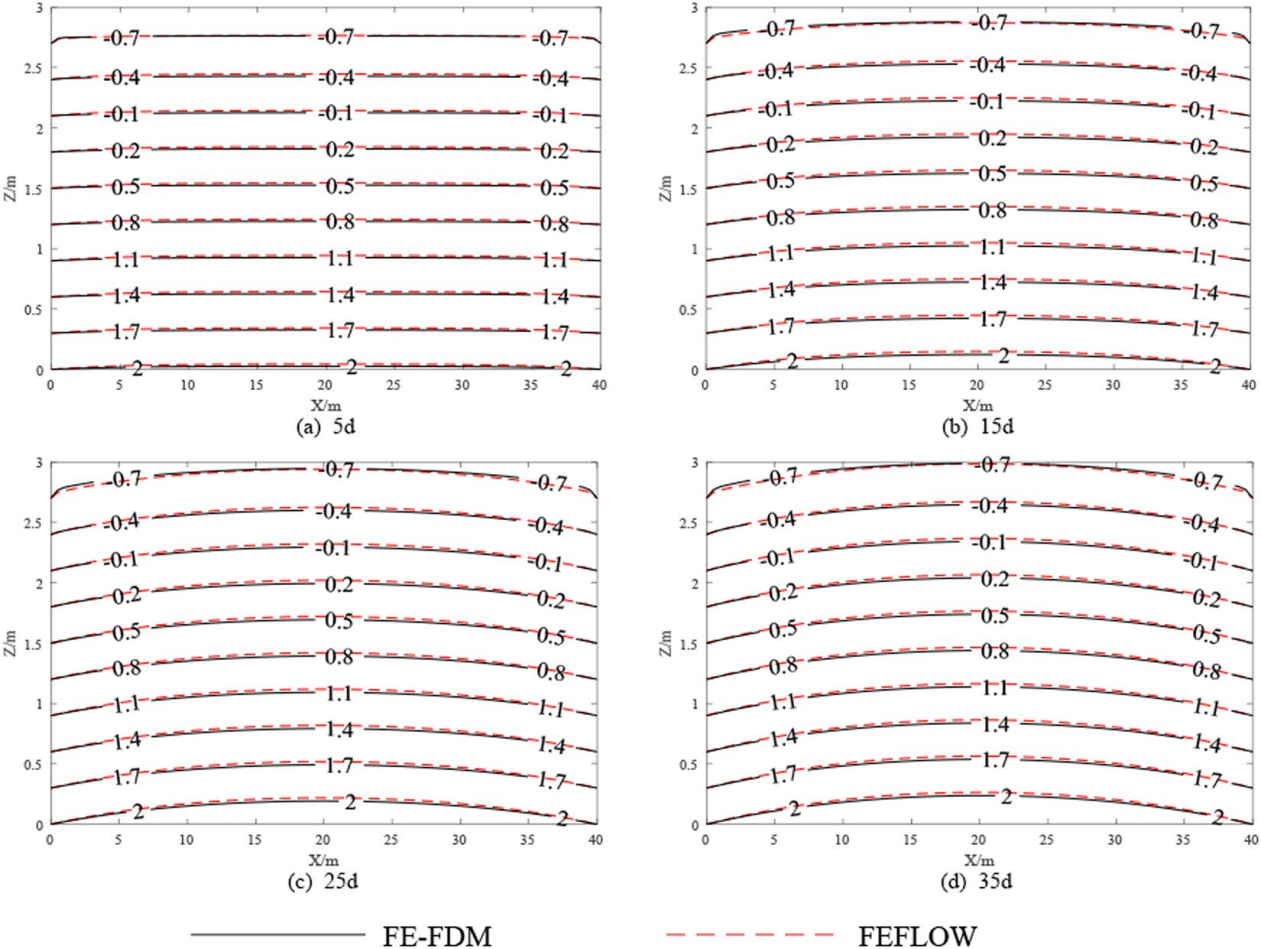
Comparison of pressure heads from FE-FDM and FEFLOW.

**Figure 4:**
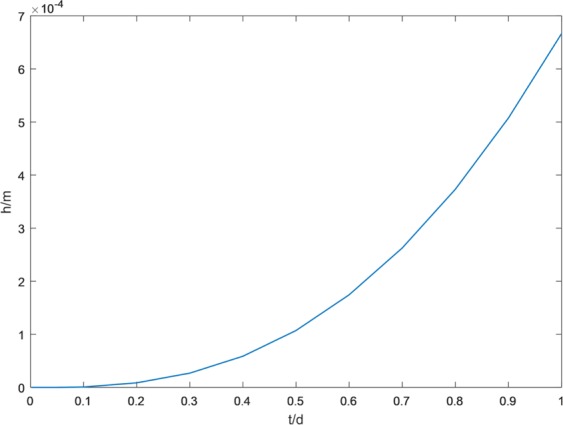
The change process of pressure heads in the location of (20,2) m.

The pressure heads calcualted by FE-FDM and FEFLOW at t = 5d, 15d, 25d and 35d are shown on Fig. 3. FE-FEDM results are in good agreement with FEFLOW solutions at the different times. At early time (i.e. t = 5d), the pressure heads are similar to initial conditions. The smaller the contour of the pressure head, such as −0.7 m, has the greater the change, since the moisture content mainly in the unsaturated zone. At t = 35d, all the pressure heads have been raised obviously. Figure 4 shows this process more clearly. In Fig. 4, pressure head change process is shown at the location of (20,2) m which is at the center of water table in the initial time. As shown in the Fig. 4, the pressure head begins to grow after about 0.1d. There is a lag time when groundwater is recharged, since infiltrated water should pass through the vadose zone. The lag time depends on the characteristics of the vadose zone.

### Test problem 2: Sand tank experiment

**Figure 5:**
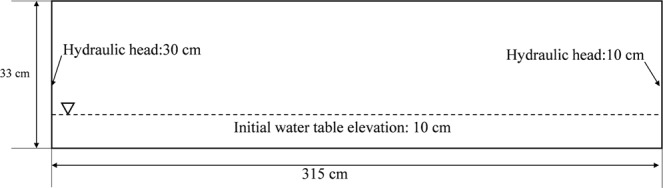
The model of test problem 2.

In this example, FE-FDM and FEFLOW are used to simulate a transient sand tank experiment which was conducted by Koichi *et al*.^27^. The sand tank is 315 cm in length, 23 cm in width and 33 cm in height. A two-dimensional vertical profile model was used to simulate the sand tank experiment. The soil parameters for the tank experiments, which are the same as those used in the model, are presented in Table 2. Figure 5 shows the conceptual model for test problem 2. The hydraulic head on the left boundary is increased from 10 cm to 30 cm at time equal zero, and the hydraulic head on the right boundary is 10 cm, both of which are constant head boundaries. The top and bottom boundaries are impermeable. The initial condition is a water table at 10 cm. The same rectangular mesh is used for the FEFLOW and FE-FDM models. The domain is discretized with 41 rows and 31 columns of nodes. The transient simulation was run for 4,800 seconds.

**Table 2:** Soil parameters of test problem 2.

Ks (cm/s)	n (1)	α (1/cm)	θs (1)	θr (1)
0.33	1.8	0.35	0.44	0.04

**Figure 6:**
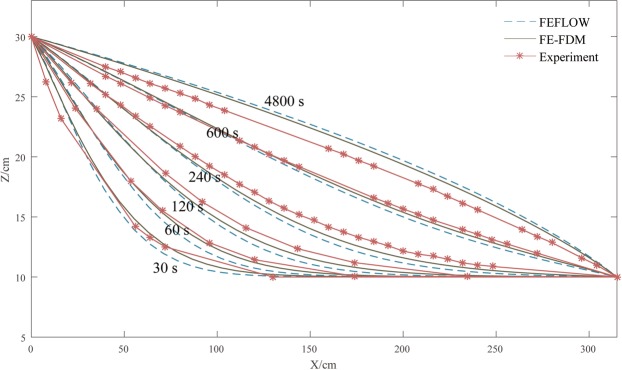
Experimental elevations of water table and water table elevations calculated with FE-FDM and FEFLOW at times ranging from 30 seconds to 4800 seconds.

Figure 6 shows the water table evaluations measured in the experiment and the water table elevations calculated with FE-FDM and FEFLOW as various times ranging from 30 seconds to 4,800 seconds. The results of FE-FDM are in good agreement with experimental data and FEFLOW. The water table elevations calculated by FE-FDM are closer to the experimental data than those calculated with FEFLOW. As shown in Fig. 6, the water tables gradually rise during the experiment.

**Figure 7:**
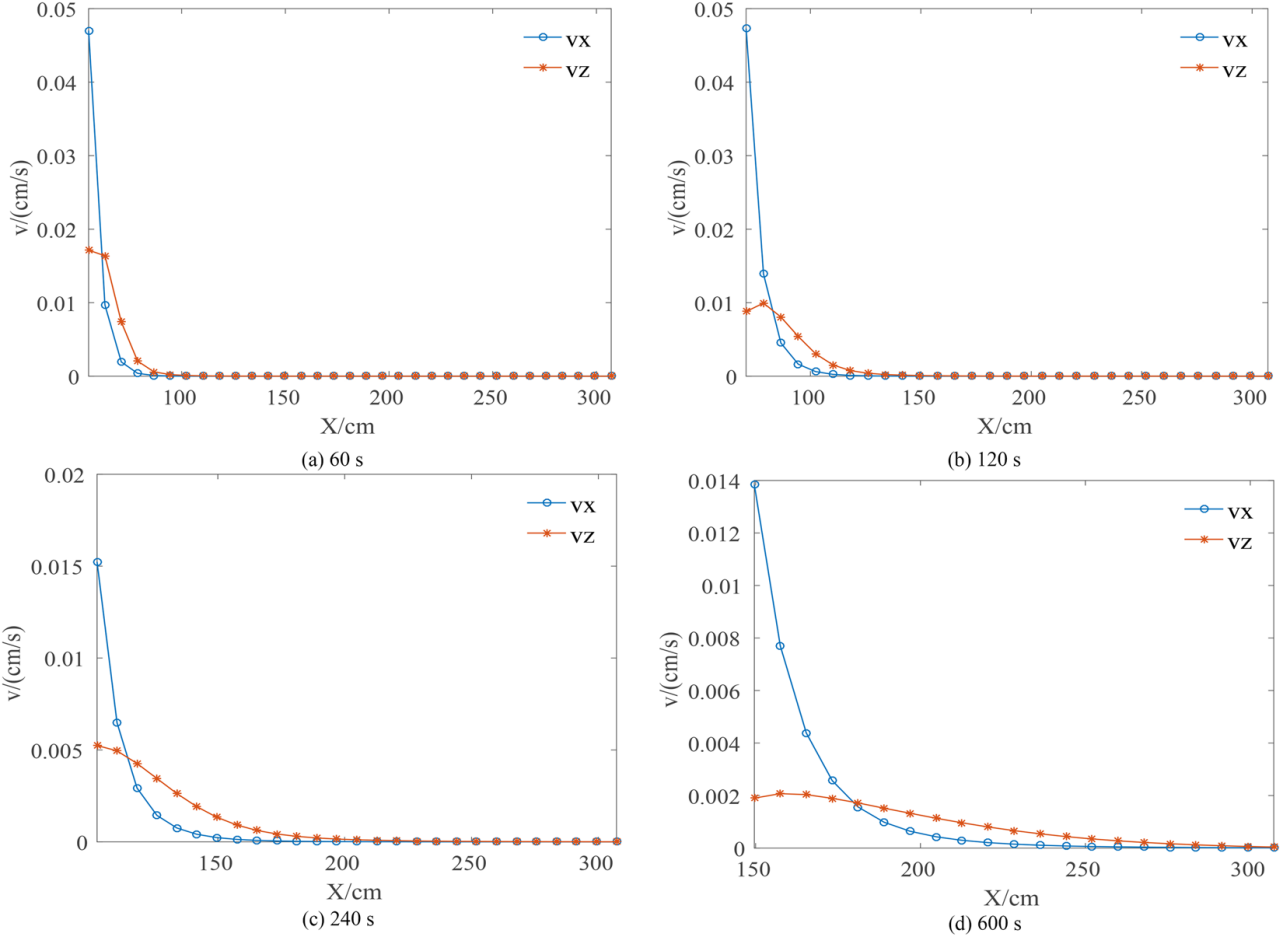
The comparison of the horizonal velocity and the vertical velocity distribution in the unsaturated zone of z = 20 cm segment.

Figure 7 shows the lateral velocity and the vertical velocity distributions in the unsaturated zone at z = 20 cm segment. As the Fig. 7 shows, both the lateral velocity and the vertical velocity decrease with distance from the infiltration line. The lateral velocity is much larger than the vertical velocity near the infiltration line. Thus the horizontal movement of water flow has an important role in the unsaturated zone where lateral flow is evident. Some numerical models ignore the horizontal velocity, because the horizontal hydraulic gradient is usually smaller than the vertical gradient^24,28,29^. This case, demonstrates why a fully 3D coupling of the saturated-unsaturated model is necessary.

### Example 3: 3D unsaturated-saturated water flow in large scale

In this example, a hypothetical large-scale region is simulated by FE-FDM. The model domain is 10,000 m × 10,000 m × 30 m. The soil parameters are presented in the Table 1. The hydraulic head is 20 m around the boundary of the model domain, which is a constant head boundary. The bottom boundary is impermeable. The recharge is 0.005 m/d at the top surface. The initial hydraulic head is 20 m in the model. Although this model is highly simplified, the model can be used to verify whether the FE-FDM can be applied to large-scale sites.

In this model, the scale in the horizontal direction is much larger than that in the vertical direction. Because hydraulic conductivity varies greatly in vertical direction in the unsaturated zone, a fine mesh spacing of 2 m is specified in the vertical direction. Course meshing is specified in the horizontal direction which has 256 triangle elements in every layer. The average side length of meshes is 954 m in the horizontal direction. The average side length of the meshes in the horizontal direction is 477 times the length in the vertical direction in this model. Therefore, the horizontal dimension is much larger than the vertical direction not only for the whole model but also for a single mesh.

**Figure 8:**
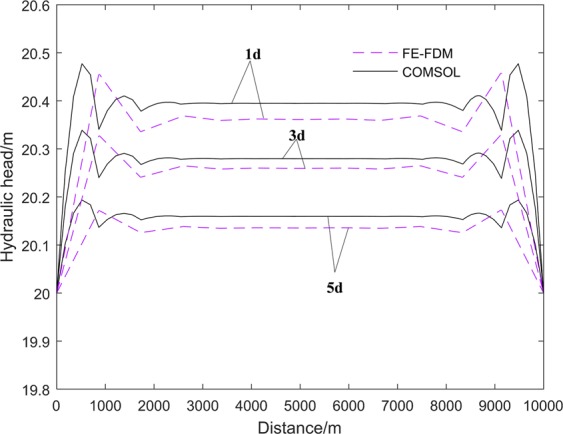
Water table elevations calculated by FE-FDM and COMSOL in the in the symmetry axis of top surface.

FE-FDM and COMSOL are used to solve this test problem. COMSOL is a multiphysical field numerical simulation software which is a stable program that covers a wide range of applications for those interested in ground water modeling based on FEM^30^. The same mesh is used for both FE-FDM and COMSOL. Figure 8 shows the water table elevations in the central section at 1d, 3d and 5d. There are differences between the values calculated by FE-FDM and COMSOL. The differences mainly are lower by 0.035 m and their ratio to COMSOL solution are mainly less than 0.2%. The changes of the water table elevations with time as calculated by FE-FDM and COMSOL are consistent. The water table elevations fluctuate near the boundary of the model as the mesh is not fine enough to accurately calculate the water table position near the boundary. It is obvious that the results of FE-FDM are less affected by the boundary than that of COMSOL. The FE-FDM has better adaptability to coarse mesh than COMSOL. This proves that FE-FDM can be applied to large scale models.

## Conclusion

In this study, a new method FE-FDM is developed to simulate and solve the saturated-unsaturated flow equation. In this method, FEM and FDM are used to solve Richards equation in the horizontal direction and in the vertical direction, respectively. The new method combines the advantages of FEM in domain discretization especially for the model boundaries and the advantages of FDM in modeling simplicity and efficiency. Three examples are used to evaluate the accuracy and numerical behavior of FE-FDM. Results of FE-FDM are compared with t experimental result and common software including FEFLOW and COMSOL. The results show that solutions of FE-FDM are accurate. The FE-FDM model has good potential for efficiently simulating complicated large-scale unsaturated–saturated water flow problems. The solving speed is not superior to general software because of the Picard iteration which has good stability but slow speed used in this paper. Future work will improve the solving speed for FE-FDM.
